# Structural basis for the inhibition of HIV-1 Nef by a high-affinity binding single-domain antibody

**DOI:** 10.1186/1742-4690-11-24

**Published:** 2014-03-13

**Authors:** Sebastian Lülf, Julie Matz, Marie-Christine Rouyez, Annika Järviluoma, Kalle Saksela, Serge Benichou, Matthias Geyer

**Affiliations:** 1 Center of Advanced European Studies and Research, Group Physical Biochemistry, Bonn, Germany; 2 Department of Physical Biochemistry, Max Planck Institute of Molecular Physiology, Dortmund, Germany; 3 Inserm U1016, Institut Cochin, Paris, France; 4 CNRS, UMR8104, Paris, France; 5 Université Paris-Descartes, Sorbonne Paris-Cité, Paris, France; 6 Department of Virology, Haartman Institute, University of Helsinki, Helsinki, Finland

**Keywords:** HIV-1 Nef, Single-domain antibody, Crystal structure, Neffin

## Abstract

**Background:**

The HIV-1 Nef protein is essential for AIDS pathogenesis by its interaction with host cell surface receptors and signaling factors. Despite its critical role as a virulence factor Nef is not targeted by current antiviral strategies.

**Results:**

We have determined the crystal structure of the complex formed by a camelid single-domain antibody fragment, termed sdAb19, bound to HIV-1 Nef together with a stabilizing SH3 domain. sdAb19 forms a stoichiometric 1:1 complex with Nef and binds to a conformationally conserved surface at the C-terminus of Nef that overlaps with functionally important interaction sites involved in Nef-induced perturbations of signaling and trafficking pathways. The antibody fragment binds Nef with low nanomolar affinity, which could be attenuated to micromolar affinity range by site-directed mutagenesis of key interaction residues in sdAb19. Fusion of the SH3 domain to sdAb19, termed Neffin, leads to a significantly increased affinity for Nef and formation of a stoichiometric 2:2 Nef–Neffin complex. The 19 kDa Neffin protein inhibits all functions of Nef as CD4 and MHC-I downregulation, association with Pak2, and the increase in virus infectivity and replication.

**Conclusions:**

Together, sdAb19 and Neffin thus represent efficient tools for the rational development of antiviral strategies against HIV-1 Nef.

## Background

The human immunodeficiency virus (HIV) is a persistent pathogen that caused an estimated 1.6 million people deaths in 2012 [1]. Of the fifteen proteins encoded by the HIV genome, the three viral enzymes, protease, integrase and reverse transcriptase are indispensable for the production of viral progeny. These enzymes are core targets of highly active anti-retroviral therapy (HAART) together with proteins mediating virus entry [2,3]. HAART allowed considerable success in reducing viral loads beyond detection levels and elongating patient life expectancy, but the current therapy is unable to clear the virus due to the persistence of latent reservoirs [4]. Advances for a successful eradication strategy showed that HAART in combination with targeted cytotoxic therapy was able to profoundly deplete productively infected cells of viral RNA [5]. In addition, many broad and potent donor-derived antibodies were uncovered in recent years, suggesting they could be valuable additions to anti-HIV-1 therapies [6]. Yet, the rapid emergence of drug resistant mutants and the increased worldwide spread of treatment resistant HIV-1 variants pose increasing problems to effective treatment of HIV-infected patients. One strategy to improve this situation is the exploitation of additional drug targets that could be added to the current regiment. Ideally, such targets comprise viral factors, since interference with host cell factors may compromise physiological functions or even viability of host cells.

Besides the structural proteins, HIV-1 encodes four accessory proteins to facilitate immune evasion and optimize conditions for virus replication [7]. The accessory *nef* gene encodes a 24–35 kDa protein that is found in all primate lentiviruses and is critical for the full pathogenic potential of these viruses [8]. Nef affects membrane trafficking in infected cells, *e.g.* by modulating the expression of surface receptors such as CD4, CD8, CD28, MHC-I and MHC-II, DC-SIGN and chemokine receptors in HIV-1 target cells [9]. In addition, Nef also affects signal transduction through interaction with cellular kinases like Pak2 and Hck to modulate signaling pathways in infected cells [9,10]. To achieve this multitude of activities, Nef has evolved as a versatile adaptor for protein interactions that lacks intrinsic enzymatic activity. The structure of HIV-1 Nef is characterized by its flexible loop regions that contain several sequence motifs as an N-terminal myristoylation site, a central poly-proline PxxP motif for SH3 domain binding and C-terminal motifs for interaction with clathrin-associated endosomal adaptor protein complexes [11].

Although compounds interfering with Nef's activity would be in multiple ways beneficial to the host, Nef is currently not a target of antiviral measures. The Nef protein is not essential for replication of HIV in the infected host, yet the protein promotes the progression to AIDS in humans by the different internalization profiles found in SIV or HIV infected cells for CD3 and CD4 T cell receptors [12]. Previously described Nef-interacting small molecular compounds bind Nef only with relatively low affinity, and display high cytotoxicity and/or interfere with only a subset of Nef interactions and functions [13,14]. The characterization of a camelid single-domain antibody fragment, termed sdAb19, which binds to HIV-1 Nef with high affinity, has provided an alternative approach to inhibit the biological activities of Nef [15]. This 12.7 kDa antibody fragment interfered with the CD4 down-regulation activity of Nef, as well as with the association of Nef with Pak2 and the accompanying actin remodeling effects. In addition, sdAb19 was shown to counteract the Nef-dependent enhancement of virion infectivity and virus replication, and to be able to rescue Nef-mediated thymic CD4^+^ T cell maturation defects in transgenic mice expressing Nef [15,16]. Here, we describe the crystal structure of the sdAb19 single domain antibody in complex with HIV-1 Nef_SF2_ and an engineered SH3 domain of Hck. We provide structural and functional evidence for the potent inhibition of Nef caused by occupation of a highly conserved surface epitope at the C-terminus of Nef. These data represent important findings for the rational development of new antiviral strategies targeting HIV-1 Nef.

## Results

### Architecture of the Nef–sdAb19–SH3_B6_ complex

**Figure 1:**
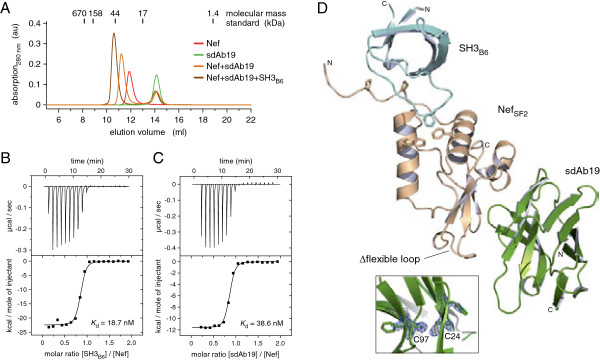
**Structure of the tripartite SH3**_**B6**_**–Nef–sdAb19 complex. (A)** Size exclusion chromatography of Nef supplemented with sdAb19 and SH3_B6_ reveals the equimolar hetero-trimeric association of the three subunits. **(B)** ITC measurement of SH3_B6_ binding to Nef_SF2_. **(C)** Binding of the camelid antibody sdAb19 to HIV-1 Nef showed a dissociation constant of 39 nM. **(D)** Crystal structure of HIV-1 Nef_SF2_ (beige) in complex with camelid sdAb19 (green) and the SH3 domain of Hck (light blue). The two Nef interacting proteins bind to opposite surfaces of Nef. The position of the C-terminal flexible loop in Nef is indicated. Cysteines C24 and C97 in the canonical fold of sdAb19 are reduced in the crystal structure as shown in the final 2*F*_*o*_*–F*_*c*_ electron density map displayed at 1 σ (inset). The PDB accession number of the tripartite sdAb19 complex is 4ORZ.

The Nef–antibody complex was formed by mixing a purified recombinant form of HIV-1 Nef_SF2_ (45–210) deleted of the first 44 N-terminal residues with sdAb19, and adding the SH3 domain of human Hck, termed SH3_B6_ and engineered for high affinity binding to Nef, to this complex [17,18]. Analytical gel filtration showed that addition of sdAb19 and SH3_B6_ to Nef_SF2_ led to formation of a stoichiometric 1:1:1 complex whose elution volume at an apparent mass of 45 kDa corresponded well to the calculated mass of 41.6 kDa (Figure 1A). To characterize the tripartite SH3_B6_–Nef–sdAb19 complex formation, we determined the individual binding affinities between Nef and its two complex partners by isothermal titration calorimetry (ITC). The two interacting domains, SH3_B6_ and sdAb19, targeted Nef with similar individual affinities, showing dissociation constants of 19 nM and 39 nM, respectively, for binding to non-myristoylated Nef_SF2_ (45–210) (Figure 1B,C). To explore if myristoylation of Nef affects binding to sdAb19, we used the lipidated protein and performed ITC measurements (Table 1). Myristoylated Nef was prepared by coexpression of full length Nef_SF2_ with the N-myristoyl transferase and addition of myristic acid to the expression media [17]. The myristoylation reaction was confirmed by ESI mass spectrometry analysis (Additional file 1: Figure S1). However, Nef myristoylation showed no effect on the binding affinity to sdAb19 (Additional file 1: Figure S2A and Table 1). A similar result was observed previously for the binding of SH3_B6_ to myrNef, which was not affected by the lipid modification of the viral protein [19].

**Table 1:** Thermodynamic parameters of isothermal titration calorimetry measurements

**Titration scheme**^**a**^	***K***_**d**_	**ΔG**	**ΔH**	**ΔS**	**T ΔS**	**n**
	**(nM)**	**(kcal/mol)**	**(kcal/mol)**	**(kcal/mol/deg)**	**(kcal/mol)**	**(**^**[..] **^**/ **_**[..]**_**)**
**a.) SH3**_**B6**_**–Nef–sdAb19 complex formation**						
SH3_B6_ to Nef_SF2_^b^	18.7	−10.55	−22.51 (±0.25)	−0.040	−11.96	0.81 (±0.005)
sdAb19 to Nef_SF2_	38.6	−10.11	−11.62 (±0.005)	−0.005	−1.51	0.81 (±0.002)
**b.) sdAb19 binding to Nef**						
sdAb19 to myrNef_SF2_	32.7	−10.20	−14.97 (±0.22)	−0.016	−4.77	0.81 (±0.006)
sdAb19 to Nef_NL4–3_	18.5	−10.37	−17.97 (±0.30)	−0.025	−7.42	0.88 (±0.007)
sdAb19 to Nef_NA7_	118	−9.45	−12.82 (±0.12)	−0.011	−3.34	0.74 (±0.004)
sdAb19 to Nef_SF2_ (∆ flex. loop)	98	−9.55	−10.71 (±0.06)	−0.004	−1.16	0.87 (±0.003)
**c.) sdAb19 mutant binding**						
sdAb19 (G102R,S103E) to Nef_SF2_	920	−8.25	−9.93 (±0.56)	−0.006	−1.68	0.9 (±0.085)
sdAb19 (D60R) to Nef_SF2_	3700	−7.40	−3.38 (±0.56)	0.014	4.02	0.74 (±0.009)
sdAb19 (triple) to Nef_SF2_	–	–	–	–	–	–
**d.) Nef**_**SF2**_** mutant binding**						
sdAb19 to Nef_SF2 _(K148E)	1700	−7.86	−8.77 (±0.26)	−0.003	−0.89	0.74 (±0.015)
sdAb19 to Nef_SF2_ (M198K)	–	–	–	–	–	–
sdAb19 to Nef_SF2 _(L202K)	–	–	–	–	–	–
**e.) Neffin binding to Nef**						
Neffin to Nef_SF2_	1.6	−12.02	−38.26 (±0.17)	−0.088	−26.23	0.66 (±0.001)
Neffin to Nef_NL4–3_	3.9	−11.49	−33.19 (±0.20)	−0.073	−21.71	0.65 (±0.002)
Neffin to Nef_NA7_	14.4	−10.72	−34.69 (±0.12)	−0.080	−23.97	0.67 (±0.001)
Neffin (triple) to Nef_SF2_	23.3	−10.41	−18.13 (±0.12)	−0.026	−7.72	1.08 (±0.004)

^a^all ITC measurements were performed at 25°C.

^b^Recombinant Nef proteins refer to: Nef_SF2_ (45–210, C59S, C210A); myrNef_SF2_ (myr2-210, C59S, C210A); Nef_NL4–3_ (41–206, C206A); Nef_NA7_ (41–206, C206A).

The tripartite protein complex of HIV-1 Nef_SF2_ (45–210, ∆158-178, deleted of the C-terminal flexible loop encompassing residues 158 to 178 of Nef_SF2_), human Hck-SH3_B6_ (residues 79–138 of human Hck) and sdAb19 (residues 1–118) was purified by gel filtration and crystallized. The 2.1 Å structure was solved by molecular replacement using the Nef–SH3_B6_ domain complex as a search model [19] (Materials and Methods, Additional file 1: Table S1). sdAb19 folds into a typical immunoglobulin domain closely resembling known llama single variable (V_HH_) structures [20-23]. The SH3_B6_–Nef–sdAb19 complex adopts an elongated shape and is formed between two subunits (chains A and B assigned to SH3_B6_ and Nef, respectively) of one asymmetric unit cell with the antibody subunit from a symmetry mate unit cell (chain C' assigned to sdAb19) (Figure 1D). The Nef–sdAb19 interface covers an average molecular surface area of 718 Å^2^, whereas Nef–SH3_B6_ covers an interface of 623 Å^2^, with no contacts formed between sdAb19 and SH3_B6_. This corresponds in total to 2,683 Å^2^ buried molecular surface area on the three proteins upon assembly into the tripartite complex. The buried interface area of sdAb19 upon binding to Nef corresponds to 12% of the total solvent accessible area of the antibody. The two cysteines C24 and C97 of sdAb19, located in close proximity on opposing β-strands B and F, were found to be reduced and did not form an intramolecular disulfide bond in the crystal (Figure 1D and Additional file 1: Figure S3).

The camelid antibody was raised by immunization of the llama with recombinant Nef protein from the HIV-1 Lai allele, residues 57–205 [15]. To further characterize the sdAb19 binding specificity to different Nef alleles, we analyzed the commonly used NL4-3 and NA7 Nef proteins, which share a sequence identity of 85.7% and 89.0% with SF2 Nef, respectively. Whereas binding to Nef_NL4–3_ was about 2-fold stronger compared to the SF2 allele, the dissociation constant of sdAb19 to Nef_NA7_ was determined to 118 nM (Additional file 1: Figure S2B,C and Table 1). The binding affinity to NA7 Nef was thus 3-fold weaker than the affinity determined for sdAb19–Nef_SF2_ complex formation. Four homologous replacements occur between NL4-3 and SF2 Nef proteins in the binding interface to sdAb19, including the notable alteration from M198 to valine [24]. Likewise, four changes are found between NA7 and SF2 Nef, of which the non-homologous change from proline at position 154 in Nef_SF2_ to alanine in Nef_NA7_ is the most prominent. Overall, these changes appear to be moderate as the affinity varies only six-fold from the tightest binding allele, NL4-3, compared to the weakest binding allele, NA7. Off note, all Nef residues in the binding interface to sdAb19 are completely identical between Bru/Lai Nef and NL4-3 Nef alleles. As the average sequence identity of Nef proteins from all HIV-1 subgroups is 84% [25], the diversity of the three analyzed Nef alleles SF2, NL4-3 and NA7 represents typical variations from the consensus sequence observed in the nature.

The C-terminal flexible loop of Nef is required for cellular trafficking functions, as *e.g.* the internalization of CD4 molecules from the cell surface. Truncation of 21 residues within this C-terminal flexible loop of Nef_SF2_ reduced the binding affinity to 98 nM suggesting a minor contribution of the flexible loop to the Nef–sdAb19 binding interaction (Additional file 1: Figure S2D). The thermodynamic parameters of the interactions and the binding stoichiometries are listed in Table 1.

### Attenuation of sdAb19 binding by mutagenesis

**Figure 2:**
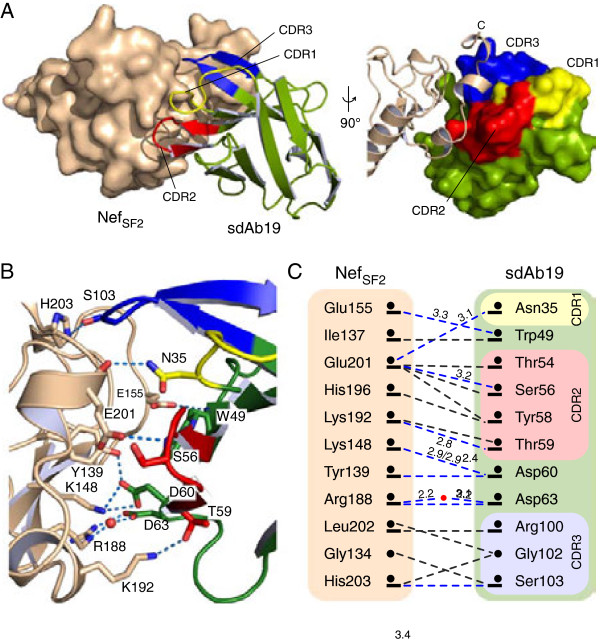
**sdAb19 targets a C-terminal surface epitope on Nef. (A)** Binding of sdAb19 to Nef_SF2_. The three complementarity determining regions are colored yellow (CDR1), red (CDR2), and blue (CDR3), respectively. **(B)** Residues of CDR2 are significantly involved in the interaction with Nef. Hydrogen bonds and salt bridges are displayed as dashed lines. **(C)** Interaction map of Nef and sdAb19 within a distance shell of 3.7 Å. Hydrophobic and polar interactions between main chain (dots) and side chain (bars) atoms are indicated by dashed lines colored grey and blue, respectively. For hydrogen bonds and salt bridges the inter-atomic distances are tabulated.

sdAb19 targets Nef mainly by its three complementarity determining regions (CDRs) (Figure 2A). The buried surface area of Nef and sdAb19 involves 43 residues according to the PDBePISA survey (http://www.ebi.ac.uk/msd-srv/prot_int/) (Additional file 1: Figure S3). Of these, eleven residues in sdAb19 and eleven residues in Nef are contacting each other within a distance shell of 3.7 Å, indicating this surface patch as a conformational epitope for sdAb19. Core interacting residues form eight direct intermolecular hydrogen bonds and five salt bridges with only one water molecule buried in the binding interface (Figure 2B). Eight of the eleven directly interacting residues in sdAb19 are located in the CDRs (Figure 2C). Only one residue, N35, of the canonical CDR1 (residues 29–35 according to the definition by Chothia et al. [26]) contributes to the interaction, whereas the majority of contacts are mediated by residues located in CDR2 (residues 54–59). The non-canonical, hyper-variable CDR3 region (residues 100–107) instead appears rather short in sdAb19, and contributes only to a lesser extent to the interaction (Additional file 1: Figure S3).

**Figure 3:**
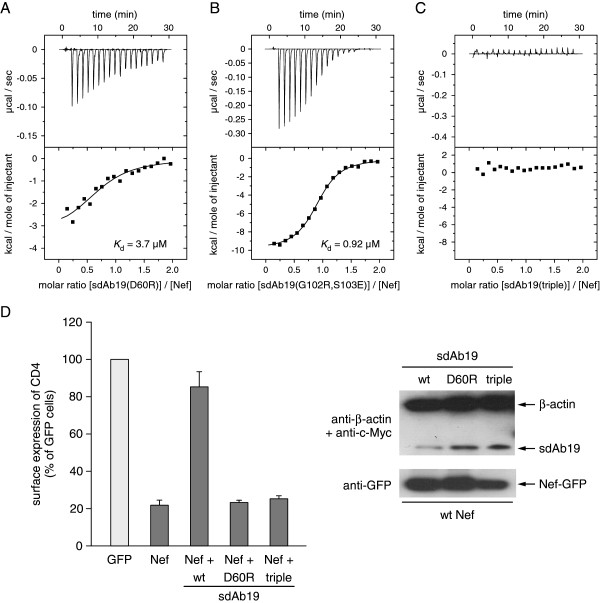
**Functional analyses of Nef–sdAb19 interactions. (A-C)** Mutation of G102R/S103E **(B)** or D60R **(A)** or the combined triple mutant D60R/G102R/S103E **(C)** in sdAb19 gradually attenuated binding to Nef as determined by ITC measurements. **(D)** Mutant sdAb19 proteins abrogate the inhibitory effect of sdAb19 on CD4 internalization. HeLa-CD4 cells were transfected with plasmids for expression of either Nef-GFP or GFP in combination with the plasmid for expression of wild-type or mutated sdAb19 (1:3 Nef:sdAb19 plasmid ratio). Transfected cells were stained with phycoerythrin-conjugated anti-CD4 at 4°C, and the surface expression of CD4 in Nef-GFP- or GFP-expressing cells was measured by flow cytometry (left panel). Results are expressed as the percentage of the mean fluorescence intensity determined in GFP-positive cells relative to that determined in GFP-negative cells. Values are the means from at least 3 independent experiments. Error bars represent 1 standard deviation from the means. Transfected cell lysates were analyzed by Western blot (right panel) using anti-c-Myc and anti-β-actin (top), or anti-GFP (bottom) antibodies.

To probe the interaction with Nef, we selected three residues in sdAb19 for site directed mutagenesis. The rational for choosing these sites was based on their location within a complementarity determining region, and the amino acid changes were designed to maximally affect the interaction without impairing the solubility of the antibody. A key residue of sdAb19 in the complex interface is D60, whose side chain carboxylic group forms ionic interactions with K148 of Nef and a hydrogen bond to Y139 (Figure 2B,C). To explore the contribution of D60 in binding to Nef, we mutated this residue to arginine, introducing thereby a charge reversal at this amino acid position while retaining the hydrophilic character of this surface residue. The dissociation constant of the sdAb19 D60R mutant for binding to Nef_SF2_ was increased to 3.7 μM as determined by ITC measurements, corresponding to a 100-fold weakening of the binding affinity (Figure 3A). In addition to this central residue, we chose two peripheral positions in sdAb19, G102 and S103, which were mutated to arginine and glutamic acid, respectively. This double mutation attenuated the binding affinity for Nef to 920 nM, corresponding to a 23-fold reduction compared to the native sdAb19 (Figure 3B). Only the combination of all three mutations, D60R/G102R/S103E, in sdAb19 finally led to a strong reduction in binding affinity, such that an interaction with Nef could no longer be detected by ITC (Figure 3C). These results showed that the binding capacity of sdAb19 to Nef can be experimentally scaled by introducing different substitutions into the key positions of this antibody fragment.

We tested the sdAb19 mutants in functional experiments for their effect on the Nef-mediated internalization of cell surface CD4. Whereas expression of Nef alone in CD4 expressing cells potently stimulated the internalization of CD4 leading to only a residual 20% CD4 expression remaining at the cell surface (Figure 3D), co-expression of sdAb19 blocked this effect and restored surface CD4 expression to levels observed in the control cells. In contrast, this capacity to counteract the effect of Nef on CD4 downregulation was lost by the D60R sdAb19 mutant and the triple-mutant D60R/G102R/S103E, further establishing the critical role of these residues for the functionality of sdAb19.

### sdAb19 targets a C-terminal surface epitope on HIV-1 Nef

**Figure 4:**
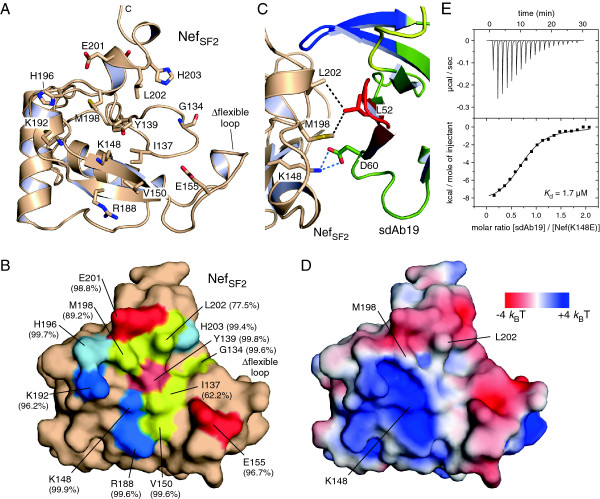
**Delineation of the sdAb19 surface binding epitope on Nef. (A)** Display of amino acids on the surface of Nef_SF2_ that interact with sdAb19. Polar residues E155, R188, K148, K192, H196, E201 and H203 surround hydrophobic residues V150, M198, L202, I137 as well as G134 and the central Y139 to constitute the binding epitope on Nef. **(B)** Surface display of the interacting residues in Nef_SF2_. Basic residues are colored blue, acidic residue are colored red, and hydrophobic residues are colored yellow. The degree of sequence conservation for homologous residues in HIV-1 Nef alleles, subtype B, is shown in brackets. **(C)** Hydrophobic interactions are formed between M198 and L202 of Nef with L52 of sdAb19 in close proximity to the K148_Nef_–D60_sdAb19_ salt bridge. **(D)** Electrostatic surface display of the binding interface for sdAb19 in HIV-1 Nef_SF2_. The electrostatic surface potential is colored from red (−4 *k*_B_T) to blue (+4 *k*_B_T). **(E)** Mutation K148E in Nef weakens the interaction by 44-fold compared to wild type SF2 Nef as determined by ITC.

The epitope on Nef that is recognized by sdAb19 encompasses a surface patch toward the C-terminus of the viral protein. A ring of charged residues, E155, R188, K148, K192, H196, E201 and H203, surrounds hydrophobic residues I137, V150, M198 and L202 at its center as well as the polar Y139 and the adjacent G134 (Figure 4A). The binding interface delineated on the surface representation of HIV-1 Nef is shown in Figure 4B. This surface patch is conserved based on the analysis of 1643 alleles of HIV-1 Nef proteins from subtype B [24]. For ten residues of the sdAb19 binding interface the degree of sequence conservation is between 96% and 99.9%, based on the analysis of homologous amino acid replacements (Figure 4B). Only three residues, M198 (89.2%), L202 (77.5%), and I137 (62.2%), share a smaller degree of sequence conservation, with the isoleucine being mostly replaced by threonine. The high degree of sequence conservation of residues in the binding interface suggests that sdAb19 binds to almost all Nef alleles of the major HIV-1 subgroups in agreement with previous results showing that sdAb19 was able to inhibit a broad panel of Nef proteins from different HIV-1 groups [15].

Three different residues in Nef, namely K148, M198 and L202, were mutated to probe their contribution to the recognition by the antibody fragment. As the corresponding residue to D60 in sdAb19, lysine 148 in Nef was mutated to glutamate in order to break the salt bridge formation by charge reversal (Figure 4C). This highly conserved lysine is in the center of a basic patch on Nef that forms the binding interface to sdAb19 as shown in the electrostatic surface display (Figure 4D). The dissociation constant for K148E increased to 1.7 μM as determined by ITC experiments, corresponding to a 44-fold weaker binding affinity compared to the wild type Nef protein (Figure 4E). Interestingly, the two-fold difference in binding affinity of the sdAb19 mutant D60R (3.7 μM) and the Nef mutant K148E (1.7 μM) could result from the different length of the two charged residues, which leads to either exposure (D60R) or retraction (K148E) of the repulsive charge.

Mutation of either residue M198 or L202 in Nef to a large, basic lysine abrogated binding to sdAb19 in both cases (Additional file 1: Figure S4), underlining the importance of hydrophobic residues at these positions. These mutants however did not impair the ability of Nef to mediate CD4 internalization as shown when expressed in CD4-positive cells (Additional file 1: Figure S5). This confirms on the one site the structural integrity of Nef upon these surface mutations. But it also suggests that residues M198 and L202 as part of the sdAb19 binding interface in Nef do not overlap with the CD4 binding surface of Nef. The inhibition of Nef-mediated downregulation of CD4 by sdAb19 might instead occur at different sites of the large interaction surface between Nef and sdAb19. Together, these results show that sdAb19 binds to a C-terminal surface epitope of Nef that overlaps with distinct functions of the viral protein.

### Neffin (sdAb19–SH3_B6_ fusion) forms a 2:2 complex with Nef

**Figure 5:**
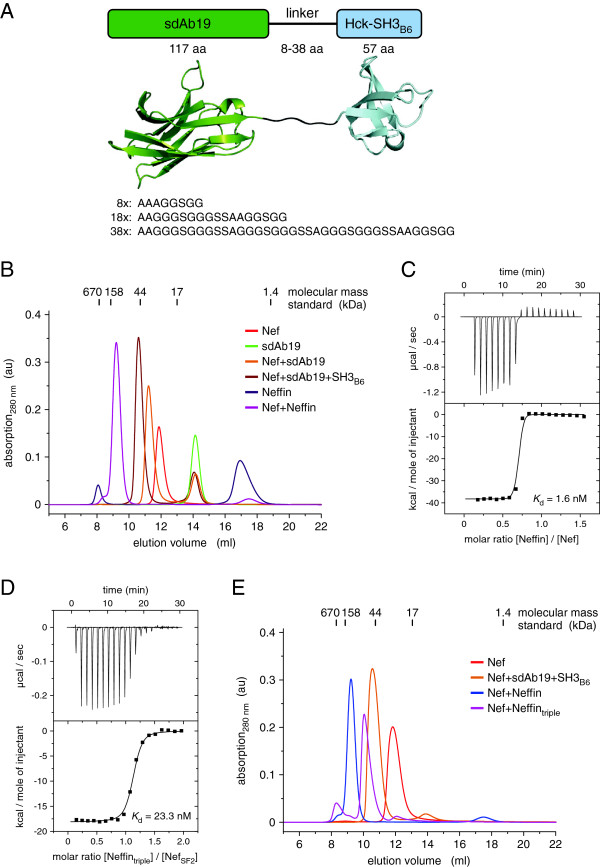
**Neffin binding to Nef results in a 2:2 complex formation. (A)** Molecular architecture of the fusion of sdAb19 with SH3_B6_ using a flexible linker of 8, 18, or 38 amino acid length. **(B)** Analytical gel filtration of Nef with sdAb19 and SH3_B6_ or Neffin (8 aa linker) reveals a significant size increase for the Nef–Neffin complex. **(C)** Isothermal titration calorimetry confirms tight binding between Nef and Neffin exhibiting a *K*_d_ of 1.6 nM. **(D)** The triple mutation D60R/G102R/S103E in the sdAb19 subunit increased the dissociation constant of Neffin_triple_ binding to Nef to the portion of the SH3_B6_ domain alone as determined by ITC measurements. **(E)** The elution volume of the Nef–Neffin_triple_ complex corresponds to an equimolar 1:1 binding stoichiometry due to the lack of sdAb19-Nef interaction.

We previously showed that fusion of sdAb19 to SH3_B6_, termed Neffin, markedly potentiated the binding affinity to HIV-1 Nef, and increased the efficacy of inhibition against all Nef functions in infected cells [16,27]. As both protein domains bind to different surface patches of Nef, the covalent linkage combined the two individual binding affinities. A scheme of the domain assembly using three different linker lengths between 8 and 38 residues is shown in Figure 5A. Unexpectedly, size exclusion chromatography revealed a significantly earlier elution volume for the Nef–Neffin complex compared to the tripartite SH3_B6_–Nef–sdAb19 complex generated from individual protein assembly (Figure 5B). This observation suggested the formation of a 2:2 Nef–Neffin complex. Variation of the linker length in Neffin up to 38 residues did not lead to formation of a 1:1 complex (Additional file 1: Figure S6), which might be explained by the opposite location of SH3 and sdAb19 binding surfaces on the structure of Nef. Of note, the very late retention of Neffin at an elution volume of 17 ml corresponds to the elution profile of the SH3 domain alone, confirming the building block construction strategy of this fusion protein. Using ITC experiments, the dissociation constant between Nef and Neffin was determined to 1.6 nM (Figure 5C). While the increase in avidity based on the accumulated strength of both subunits might not be as high as observed in other cases, *e.g.* compared to the 4,000-fold increase seen for the multivalent combination of two V_HH_ fragments [28], it should be noted that the affinity determined by ITC is at the lower resolution limit of this technique. Surface plasmon resonance of the Nef_NL4–3_–Neffin interaction showed indeed binding in the picomolar affinity regime with very low dissociation rates [27].

To analyze the individual contributions of the two Neffin subunits to the complex formation with Nef, we introduced the sdAb19 triple mutation D60R/G102R/S103E to Neffin, named Neffin_triple_. This mutation reduced the binding affinity of Neffin_triple_ to Nef to the contribution of the SH3_B6_ domain alone (Figure 5D and Table 1), in line with the previous observation that the triple mutation in sdAb19 alone abrogated the interaction with Nef (Figure 3C). Size exclusion chromatography confirmed indeed formation of a 1:1 Nef–Neffin_triple_ complex taking the expected higher hydrodynamic volume of the complex by the unbound sdAb19 subunit into account (Figure 5E).

**Figure 6:**
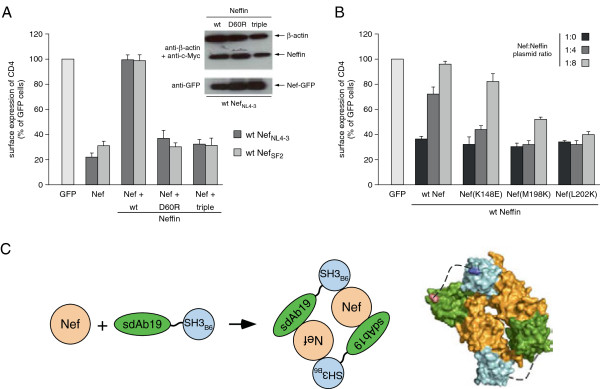
**Binding of sdAb19 in Neffin correlates with the inhibition of CD4 internalization. (A)** HeLa-CD4 cells were transfected with plasmids for expression of either Nef-GFP or GFP in combination with the plasmid for expression of wild-type or mutated sdAb19 (1:3 Nef:sdAb19 plasmid ratio). Transfected cells were analyzed for CD4 cell surface expression and cell lysates were analyzed by Western blotting (inset panels) as in Figure 3D. **(B)** HeLa-CD4 cells were transfected with plasmids for expression of either Nef-GFP, mutant Nef-GFP or GFP alone (1:0 plasmid ratio) or in combination with increasing amounts of the plasmid for the expression of wild-type Neffin (1:4 or 1:8 plasmid ratio) and analyzed for CD4 cell surface expression. **(C)** Model of the complex formation between Nef and Neffin, consisting of the two individual Nef-binding domains sdAb19 and SH3_B6_. Formation of a stable 2:2 complex leads to coverage of additional surfaces in Nef that increases the inhibitory potential of Neffin against Nef functions.

Neffin_triple_ as well as the Neffin D60R mutant were tested in functional experiments to study their contribution to the impairment of the Nef-induced CD4 internalization (Figure 6A). Whereas wild-type Neffin potently inhibited CD4 down-regulation by Nef, and fully restored CD4 surface expression levels for both NL4-3 and SF2 Nef alleles, the D60R mutant and the triple mutant failed to block CD4 down-regulation. This indicates that SH3 binding alone does not affect Nef's ability to interact with CD4 and connect it to the intracellular trafficking machinery, in line with previous observations [29]. Likewise, mutation of K148E, M198K or L202K in Nef, which strongly reduced binding to sdAb19, showed a gradually reduced susceptibility for the inhibition of CD4 internalization by wild-type Neffin (Figure 6B). These data indicate a reciprocal correlation between sdAb19 binding and the ability of Nef to downregulate CD4. Nef(K148E), which was still able to bind sdAb19 but with a lower affinity (see Figure 4E), was functionally inhibited by Neffin only when high levels of Neffin were co-expressed at a 1:8 Nef:Neffin expression plasmid ratio. In contrast, the M198K and L202K mutants, which failed to display any affinity for sdAb19 (Additional file 1: Figure S4), were not or only poorly inhibited by Neffin even at the highest concentration (Figure 6B). Therefore, the formation of the 2:2 Nef–Neffin complex may lead to an additional coverage of Nef surfaces that would be otherwise accessible for interactions with host cell factors (Figure 6C). This effect might additionally contribute to the potency of Neffin for the inhibition of all HIV-1 Nef functions.

## Discussion

Here we define the structural basis of HIV-1 Nef inhibition by the camelid-derived sdAb19 antibody fragment. sdAb19 binds to a C-terminal surface epitope on Nef that overlaps with multiple interaction sites of the viral protein. Nef was shown to target the Pak2 serine/threonine kinase by a C-terminal sequence motif that involves K192 and F195 residues [30]. We find that K192 and H196 in Nef are directly interacting with sdAb19. These overlapping surface interaction sites may thus well explain how the interaction of Nef to Pak2 is impaired by the tight binding of sdAb19 [16], although other residues as the N-terminal VGF motif were shown to affect Pak2 binding as well [31].

The interaction with the cytoplasmic internalization motifs of CD4 and other T cell surface proteins has been mapped to a hydrophobic sorting motif recognition site between the two central helices in the core domain of Nef [32-34]. Although sdAb19 does not directly interact with residues of Nef that are supposed to mediate the interaction with CD4, its close proximity to the C-terminal flexible loop is likely involved in inhibition of this function. The C-terminal flexible loop of Nef harbors a di-leucine based sorting motif as well as flanking acidic motifs whose presence is required for Nef trafficking functions via contacts with the clathrin-associated adaptor protein machinery [35,11]. Deletion of the central 21 residues in the flexible loop of Nef reduced the binding affinity to sdAb19 by three-fold. As sdAb19 directly interacts with E155 in the flexible loop of Nef, this could affect the interaction of Nef with the endocytic machinery, thus inhibiting the downstream effects of Nef on cell surface receptor internalization. In line with this suggestion we previously showed that sdAb19 disrupted the direct interaction of Nef with endosomal adaptor protein complexes [16]. The antibody fragment however had no effect on the subcellular localization of Nef as previously shown [15]. This observation is in line with the finding that sdAb19 shows similar binding affinities for an N-terminally truncated Nef 45–210 variant as well as the myristoylated full length protein. The data confirm that the myristate and the N-terminal polybasic patch that sustains membrane binding [36-39] are free to interact with lipid compartments even in the Nef–sdAb19 complex. These observations might explain the inhibition of all internalization stimulating functions of Nef by sdAb19 except for the downregulation of MHC-I, which supposedly does not occur at the plasma membrane [40]. This additional function is only abrogated through the coverage of the PxxP motif and flanking residues on the core domain of Nef by the SH3 domain moiety of Neffin [16].

The distinct 2:2 stoichiometry of the Nef–Neffin complex formation is surprising given that the two constituting domains, sdAb19 and SH3_B6_, bind Nef with similar affinities. While the topology of the interaction as seen from the structure determination clearly shows how both molecules bind to opposing sites of Nef separated by a long distance, it is perhaps unexpected that we did not observe strings of Nef–Neffin assemblies, where high aggregates would form through alternating domain interactions. Such aggregation strings would occur if the second Neffin molecule binding to a preformed Nef–(sdAb19-SH3_B6_)–Nef complex would recruit a third Nef subunit into this complex. Instead, the sharp elution profile at a 2:2 molecular mass suggests the specific and very tight quaternary Nef–Neffin complex formation. It seems reasonable to propose that such an assembly could additionally contribute to the inhibitory function of Neffin, exceeding the combined effects of sdAb19 and SH3 alone, as additional surfaces of Nef might be covered through the 2:2 complex formation. A model regarding how a 2:2 Nef–Neffin complex assembly could lead to additional coverage of Nef surfaces is illustrated in Figure 6C.

## Conclusions

The structural and functional characterization of sdAb19 and Neffin binding to Nef opens a broad avenue for their rational usage. sdAb19 and Neffin can be used (*i*) for basic research as biochemical tools for in vitro analysis at the molecular and cellular level, where they could impair intracellular signaling and trafficking pathways mediated by Nef in infected cells. (*ii*) They can also be used for in vivo evaluation of inhibition of the dysregulatory effects of Nef on the immune system in a relevant animal model such as HIV-1 infected “humanized” mice. These latter experiments should also give definitive answer regarding validation of Nef as a rational target for development of new antiretroviral strategies. (*iii*) sdAb19 can be used as a tool for small chemical compound identification or lead compound optimization in high throughput assays or drug design approaches based on the structure of the Nef–sdAb19 complex. Through binding to the molecular area recognized by sdAb19, these compounds could mimic the inhibitory activity of sdAb19 on Nef functions. (*iv*) sdAb19 or Neffin might ultimately be directly used in HIV treatment through expression in infected cells either by gene transfer or after development of nano- and microparticle-based formulation strategies for efficient intracellular delivery. As both sdAb19 and Neffin proteins can be produced in high amounts in *E. coli*, their easy and low cost production and the possibility to tune their binding affinity to Nef from the sub-nanomolar to the high micromolar affinity range might facilitate a broad application in both academic and pharmaceutical research.

## Methods

### Plasmid cloning and protein production

Bacterial expression plasmids for HIV-1 Nef_SF2_, the SH3 domain of human Hck, and llama sdAb19 were described previously [19,27,41]. Expression and purification of full length myristoylated Nef_SF2_ protein (myr2-210, C59S, C210A), an N-terminal deletion construct of Nef_SF2_ (45–210, C59S, C210A), and a Nef_SF2_ variant containing a deletion of the C-terminal flexible loop (45–210, ∆158-178, C59S, C210A) was performed similarly as described [17,41]. sdAb19 (1–118) was expressed as GST-fusion protein in *E.coli* and purified by affinity chromatography as described [27].

### Nef_SF2_–sdAb19–SH3_B6_ crystallization

Initial screening for crystallization conditions of the Nef_SF2_–sdAb19–SH3_B6_ complex was carried out using a Mosquito robot (TTP Labtech) with the sitting-drop method at 293 K and a concentration of 5–15 mg/ml. About 0.1 μl of protein solution was mixed with 0.1 μl of reservoir solution from a 70 μl reservoir in 96-well Hampton 3553 crystallization plates. Initial crystals of Nef_SF2_–sdAb19–SH3_B6_ could be obtained in 0.2 M potassium formate and 20% polyethylene glycol (PEG) 3350. Crystal conditions were optimized to 0.2 M potassium formate, 17.5% polyethylene glycol (PEG) 3350 and 0.35 M ammonium chloride grown by hanging-drop vapor diffusion in Linbro crystallization plates. Crystals grew under these conditions within 12 days to a size of 250 × 40 × 40 μm. For cryo-protection, crystals were transferred to a solution that contained the reservoir buffer with additional 25% ethylene glycol. After 5–10 seconds, crystals were flash-cooled in liquid nitrogen.

### Structure determination

The native diffraction data were measured from crystals cooled to cryogenic temperature of 100 K and were recorded on beamline X10SA (PXII) of the Swiss Light Source (SLS) equipped with an MAR 225 CCD detector (oscillation width per frame, 1°; 400 frames collected) to 2.1 Å resolution. The Nef_SF2_–sdAb19–SH3_B6_ crystals were of space group P4_1_ and had unit cell parameters of a = 73.07, b = 73.07 and c = 71.25 Å. Assuming the presence of one tripartite complex in the asymmetric unit, the solvent content of the crystals is 52.02%. The XDS package [42] was used to process, integrate, and scale the collected data. The structure was solved with molecular replacement using the Nef_SF2_–SH3_B6_ (PDB entry 3RBB [19]) as a search model in PHASER [43]. The model was refined by alternating cycles of manual rebuilding in COOT [44] and minimization in REFMAC5 [45]. Data collection statistics and refinement parameters are given in Additional file 1: Table S1. Protein interfaces and accessible surface areas were calculated with the program PISA (http://www.ebi.ac.uk/pdbe/). Molecular diagrams were drawn using PyMOL (http://www.pymol.org/).

### Size exclusion chromatography

Analytical gel filtrations of recombinant Nef_SF2_, SH3_B6_, sdAb19, Neffin, and all mutants thereof were performed using a multicomponent Waters 626 LC system (Waters, MA) equipped with a Superdex S75 (10/300 GL) column (GE Healthcare). Typically, 100 μl of a 1.5 mg/ml protein solution was loaded onto the column that was equilibrated in 10 mM Tris/HCl (pH 9.0), 100 mM NaCl buffer prior to injection of the protein samples. Gel filtrations were run at a flow rate of 0.5 ml per minute in 10 mM Tris/HCl (pH 9.0), 100 mM NaCl onto the S75 column at 4°C or 20°C. The optical density was monitored at a wavelength of 280 nm over the time course of the experiment. Gel filtration experiments were performed repeated times.

### Isothermal titration calorimetry

Interaction of HIV-1 Nef_SF2_ with SH3_B6,_ sdAb19 or Neffin was performed by isothermal titration calorimetry using a MicroCal iTC200 microcalorimeter (GE Healthcare). Measurements were carried out in 20 mM Tris/HCl buffer (pH 9.0), 100 mM NaCl at 25°C. SH3_B6,_ sdAb19 or Neffin at a concentration of 200 μM were stepwise injected from the syringe to 20 μM Nef_SF2_ placed in the measurement cell. The change in heating power was observed over the reaction time until equilibrium was reached. Data were analyzed using the software provided by the manufacturer.

### Cell culture, transfection, flow cytometry, and immunoblot analysis

Plasmids for expression in mammalian cells of wild-type Nef fused to the green fluorescent protein (Nef-GFP), as well as wild-type Myc-tagged forms of sdAb19 and Neffin, have been described previously [15,16]. Plasmids for expression of Nef, sdAb19 and Neffin mutants were constructed by PCR-mediated site-directed mutagenesis using specific primers and the wild-type expression vectors as templates. Cell culture experiments were performed in HeLa cells stably expressing CD4 similarly as described [15,16]. CD4 cell surface staining and flow cytometry analysis were performed as described previously [15]. Protein expression was analyzed on transfected cell lysates by Western blot using anti–c-Myc (9E10; Roche) or anti-GFP (sc-8334; Santa Cruz Biotechnology Inc.) antibodies [16].

### Accession numbers

The atomic coordinates and structure factors of the HIV-1 Nef_SF2_–Hck_SH3-B6_–sdAb19 complex have been deposited in the Protein Data Bank under accession code 4ORZ.

## Abbreviations

CDR: Complementarity determining region; GFP: Green fluorescent protein; HAART: Highly active anti-retroviral therapy; HIV: Human immunodeficiency virus; MFI: Mean fluorescence intensity; Nef: Negative factor; Neffin: Nef inhibitor; sdAb19: Single domain antibody 19; SIV: Simian immunodeficiency virus; TCR: T-cell receptor.

## Competing interests

The authors declare that they have no competing interests.

## Authors’ contributions

SL purified proteins, performed binding studies, crystallized the protein complex and determined the crystal structure. JM and MCR performed cell surface expression experiments under the supervision of SB. AJ and KS contributed reagents and expertise. KS, SB and MG analyzed data. MG designed of the study and wrote the manuscript. All authors discussed the results and commented on the manuscript. All authors read and approved the final manuscript.

## Supplementary Material

Additional file 1 **Table S1.** Data collection and refinement statistics of the SH3_B6_–Nef–sdAb19 crystal structure. **Figure S1.** Analytical characterization of HIV-1 Nef myristoylation. **Figure S2.** Isothermal titration calorimetry measurements of sdAb19 titrated to HIV-1 Nef proteins of different virus strains. **Figure S3.** Sequence and secondary structure display of the SH3_B6_–Nef_SF2_–sdAb19 complex structure. **Figure S4.** ITC measurements of sdAb19 titrated to Nef mutants. **Figure S5.** Cell surface down-regulation of CD4 is not changed by Nef mutations that affect sdAb19 binding. **Figure S6.** Size exclusion chromatography of Neffin constructs and complex formation with Nef. **Additional references.** Click here for file
