# Supplementary material for: Structural basis for the inhibition of HIV-1 Nef by a high-affinity binding single-domain antibody
Source: Retrovirology. 2014 Mar 13;11:24. doi: 10.1186/1742-4690-11-24 (PMC4007562; doi:10.1186/1742-4690-11-24)
Supplement: Additional file 1 — Table S1. Data collection and refinement statistics of the SH3B6–Nef–sdAb19 crystal structure. Figure S1. Analytical characterization of HIV-1 Nef myristoylation. Figure S2. Isothermal titration calorimetry measurements of sdAb19 titrated to HIV-1 Nef proteins of different virus strains. Figure S3. Sequence and secondary structure display of the SH3B6–NefSF2–sdAb19 complex structure. Figure S4. ITC measurements of sdAb19 titrated to Nef mutants. Figure S5. Cell surface down-regulation of CD4 is not changed by Nef mutations that affect sdAb19 binding. Figure S6. Size exclusion chromatography of Neffin constructs and complex formation with Nef. Additional references. [file 1742-4690-11-24-S1.pdf]

Additional file 1

Table S1: Data collection and refinement statistics of the $SH3_{B6}$–Nef–sdAb19 crystal structure.

Data collection and refinement statistics	
Data collection	
Beam line	SLS X10SA
Wave length (Å)	0.979400
Space group	$P4_1$ (76)
Unit cell dimensions	
a, b, c (Å)	a=b=73.07; c=71.25
α, β, γ (°)	α=β=γ=90
Resolution range (Å)	41.75-2.1
No. images	1-400
No. reflections	95,957
Unique reflections	47,918
Completeness (%)	98.94
Multiplicity	2.0
R_{sym}	7.9
Mean I/σI	17.16
Matthews coefficient ($Å^3$/Da)	2.56
Solvent content (%)	52.02
Refinement	
No. of atoms	2455
No. of water molecules	125
R_{work} (%)	20.18
R_{free} (%)	23.64
R.m.s. deviations	
Bond lengths (Å)	0.008
Bond angles (°)	1.288
<B> overall	2.97
Ramachandran plot	
Most favored (%)	93.59
Allowed (%)	6.05
Disallowed (%)	0.36
Missing amino acids	35
Accession code	
PDB accession code	4ORZ

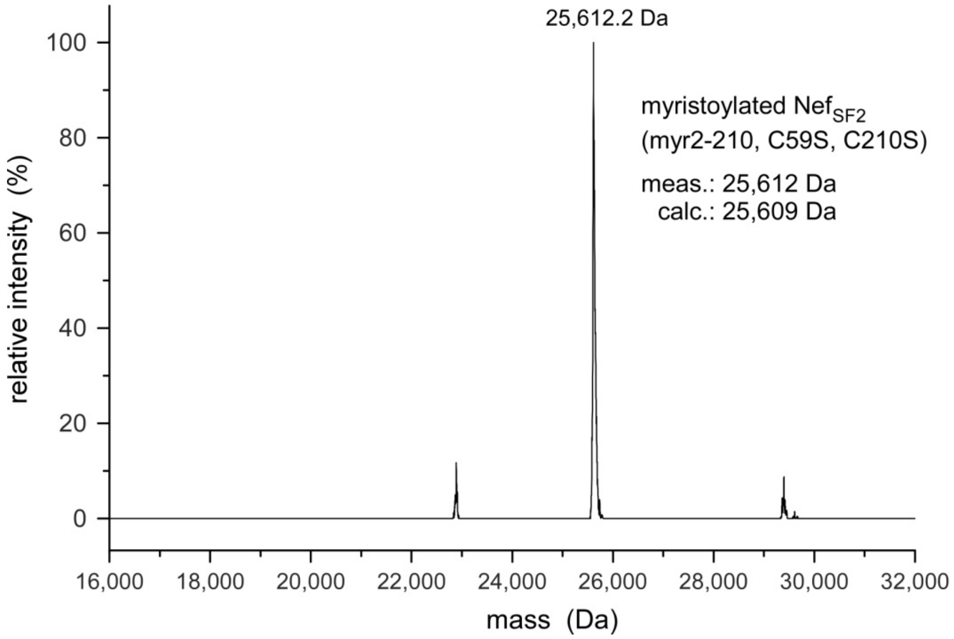

Figure S1. Analytical characterization of HIV-1 Nef myristoylation. ESI mass spectrometry analysis of the myristoylated HIV-1 Nef$_{SF2}$ protein (myr2-210, C59S, C210S) attached with a C-terminal hexa-histidine tag. The measured protein mass of 25,612 Da matches well with the calculated mass of 25,609 Da. Note, that no indication for a non-myristoylated Nef protein variant was observed, whose mass would be 228 Da lighter than the myristoylated form of Nef. This confirms the quantitative myristoylation of all Nef protein by the N-myristoyl transferase, similarly as observed before by NMR spectroscopy and Western blot analysis (Breuer et al., 2006).

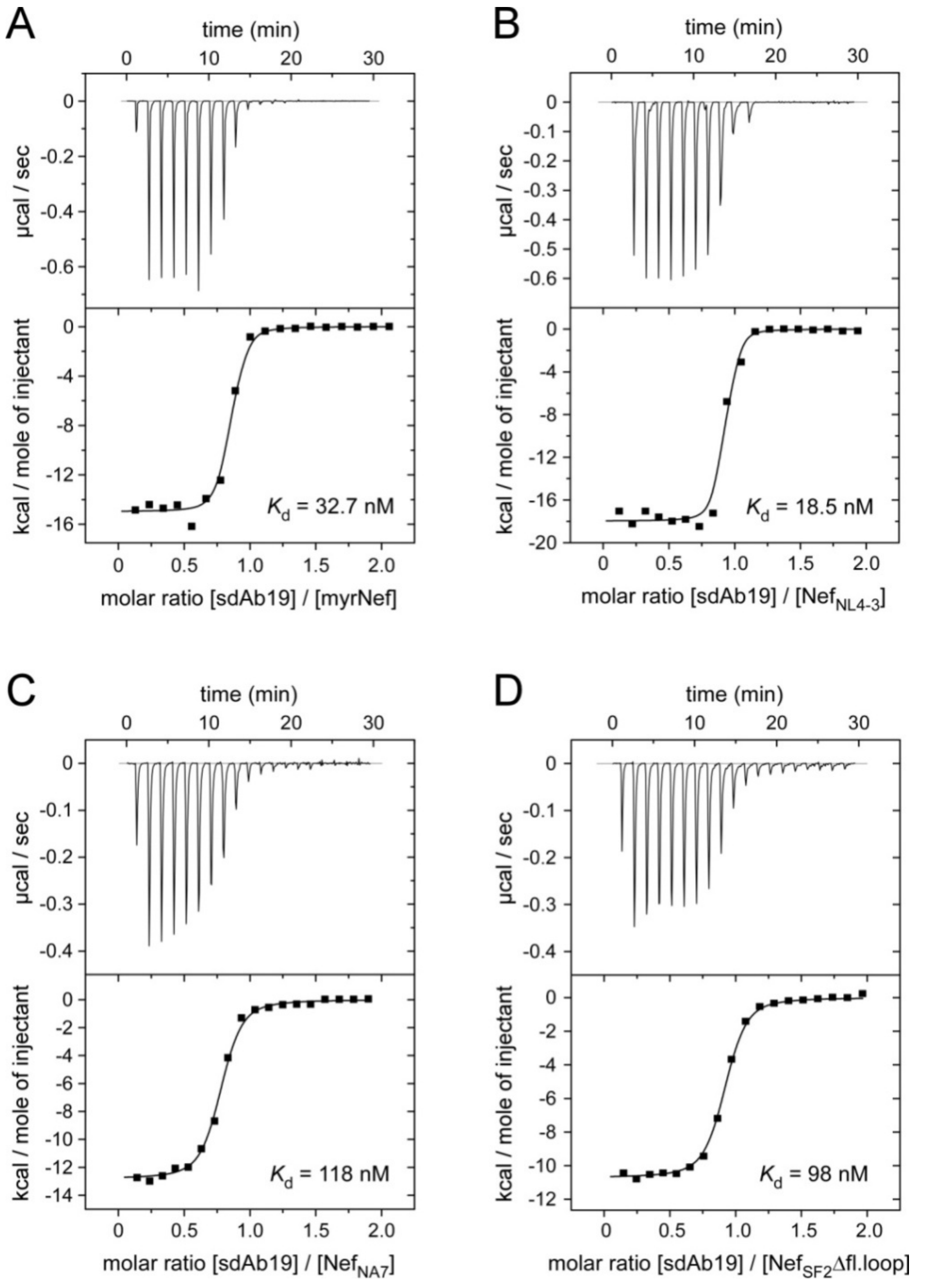

Figure S2. Isothermal titration calorimetry measurements of sdAb19 titrated to HIV-1 Nef proteins of different virus strains. (A) The camelid antibody sdAb19 interacts with a dissociation constant of 33 nM to the native myristoylated Nef protein. (B) Binding to Nef_{NL4-3} was increased by two-fold to 19 nM compared to Nef_{SF2}. (C) Nef from allele NA7 showed a reduced binding affinity of 118 nM for sdAb19. (D) Deletion of the flexible loop in the Nef_{SF2} (Nef_{SF2} 45-210,Δ158-178) reduced the binding affinity by more than 2-fold compared to the non-truncated variant. All thermodynamic parameters are listed in Table 1.

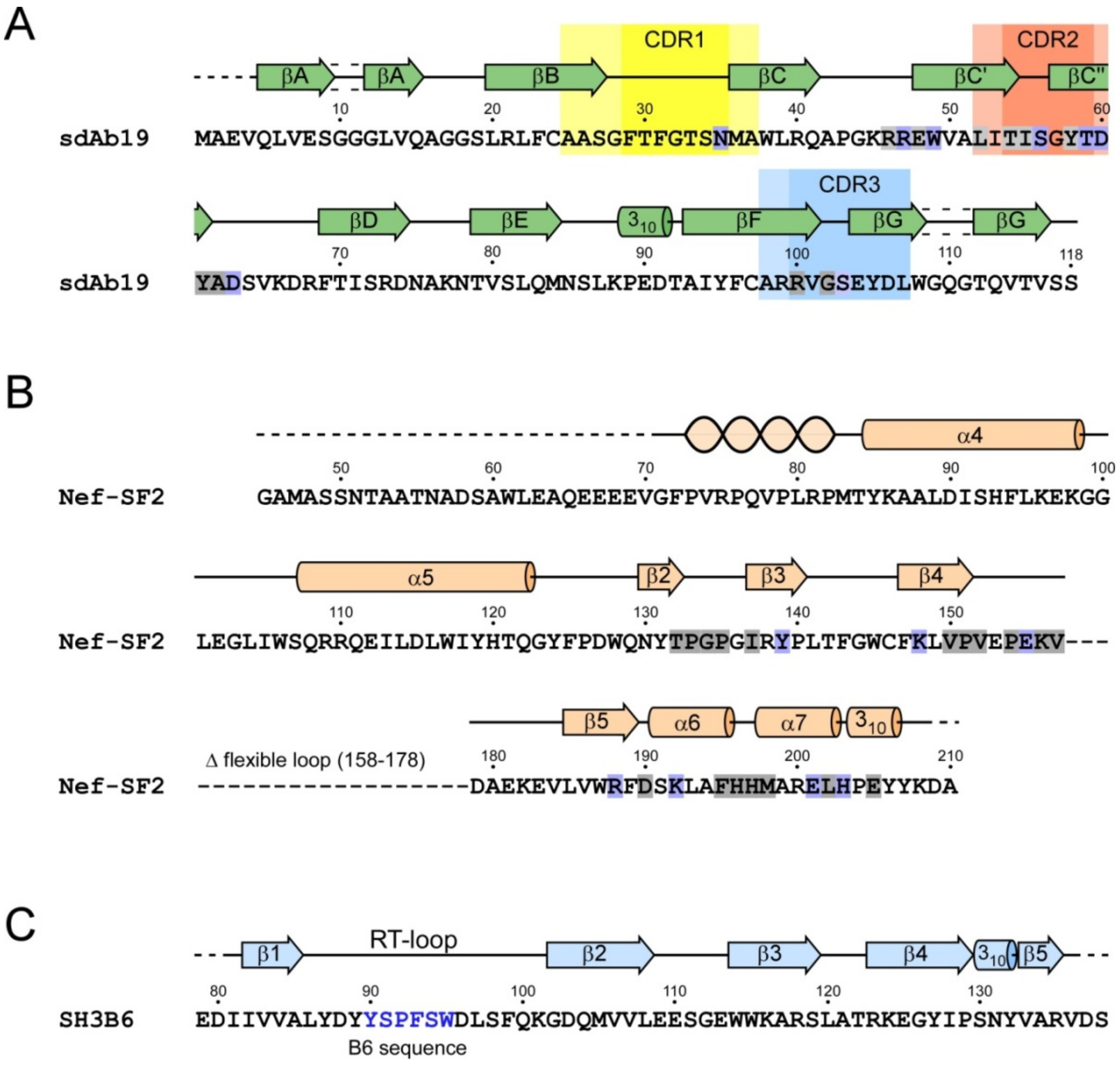

Figure S3. Sequence and secondary structure display of the sdAb19–Nef$_{SF2}$–SH3$_{B6}$ complex structure. (A) sdAb19. The three complementarity-determining regions (CDRs) according to the definition by Chothia et al., 1992, (full color) or the analysis by North et al., 2011, (light color) are indicated in yellow, red and blue, respectively. Residues whose accessible surface area is buried upon complex formation with Nef (as determined by PDBePISA) are boxed. Residues that form hydrogen bonds and salt bridges with Nef are colored blue. (B) HIV-1 Nef$_{SF2}$. Residues whose accessible surface area is buried upon complex formation with sdAb19 are colored similarly as for sdAb19. (C) Sequence and secondary structure display of human Hck SH3$_{B6}$ (79-138) in the sdAb19–Nef$_{SF2}$–SH3$_{B6}$ complex. Dotted lines in the secondary structure display above the amino acid sequence indicate invisible segments in the crystal structure.

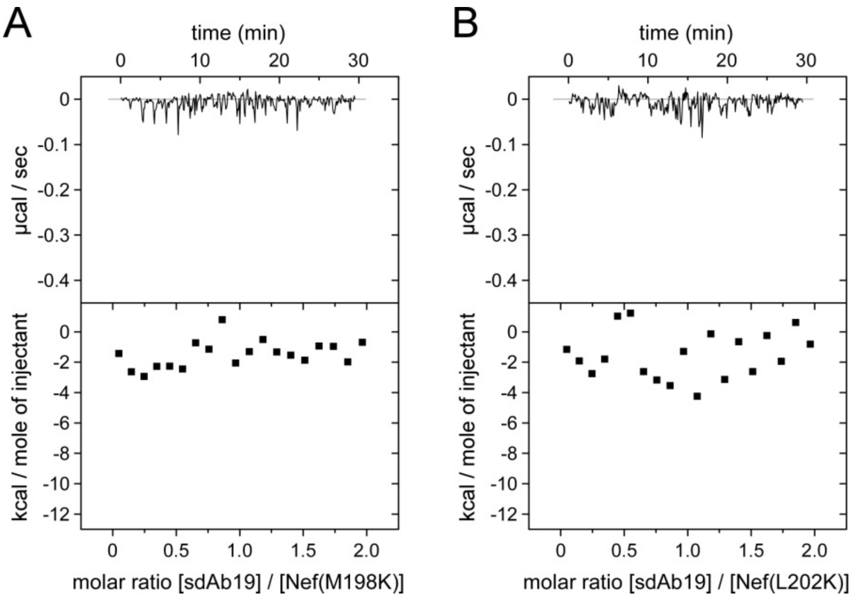

Figure S4. ITC measurements of sdAb19 titrated to Nef mutants. Single point mutations M198K (A) and L202K (B) in HIV-1 Nef_{SF2} abrogate binding to sdAb19 in isothermal titration calorimetry measurements indicating that the dissociation constant is at least weaker than 50 µM.

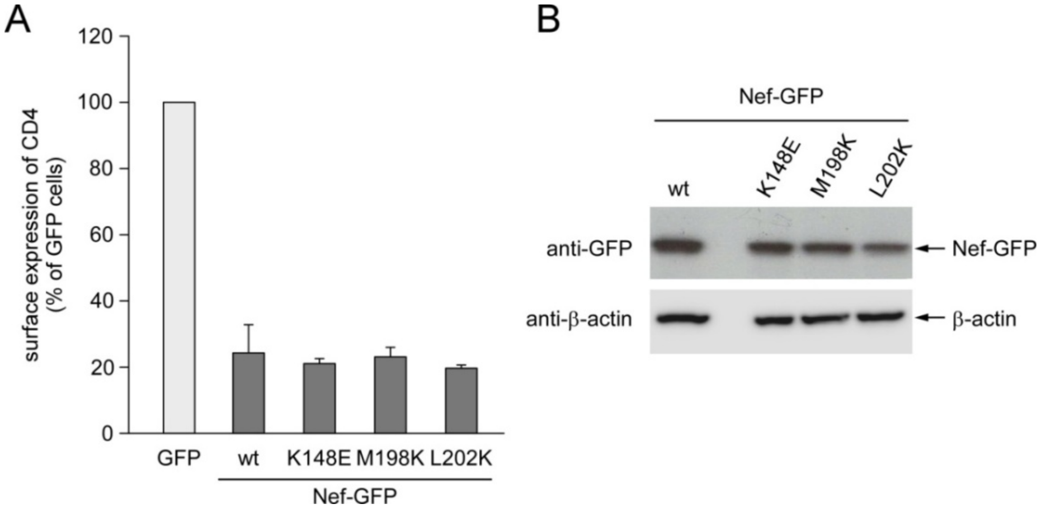

Figure S5. Cell surface down-regulation of CD4 is not changed by Nef mutations that affect sdAb19 binding. (A) HeLa-CD4 cells were transfected with plasmids for expression of wild-type or mutated Nef-GFP or GFP, and were then analyzed for CD4 cell surface expression as indicated in Fig. 4C. (B) Lysates from transfected cells were also analyzed by Western blot using anti-GFP (top) and anti-β-actin (bottom) antibodies.

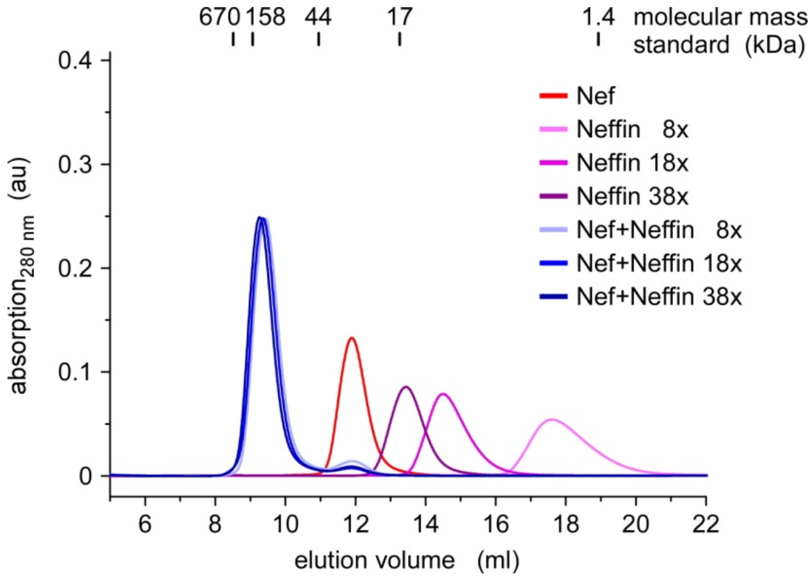

Figure S6. Size exclusion chromatography of Neffin constructs and complex formation with Nef. Neffin fusion proteins with linker lengths of 8, 18, or 38 amino acids were applied to analytical gel filtration and compared upon complex formation with Nef. Whereas the elongated linker led to an increased molecular size of Neffin proteins indicated by the earlier elution volume, the hydrodynamic size of the Nef–Neffin complexes did not change much with the lengthened Neffin proteins. This suggests that the stoichiometic 2:2 complex is maintained even for the Neffin variant with a 38 residues linker.

Additional References

Breuer S, Gerlach H, Kolaric B, Urbanke C, Opitz N, Geyer M: **Biochemical indication for myristoylation-dependent conformational changes in HIV-1 Nef.** *Biochemistry* 2006, **45:**2339–2349.

Chothia C, Lesk AM, Gherardi E, Tomlinson IM, Walter G, Marks JD, Llewelyn MB, Winter G: **Structural repertoire of the human VH segments.** *J Mol Biol* 1992, **227:**799–817.

North B, Lehmann A, Dunbrack RL, Jr.: **A new clustering of antibody CDR loop conformations.** *J Mol Biol* 2011, **406:**228–256.
